# Intemperance in Use of Quinine

**Published:** 1871-12

**Authors:** 


### Intemperance in Use of Quinine.

A newspaper says: There are various kinds of intemperance, and dosing is by no means the least of them. We are told that the amount of quinine sent from Philadelphia to the West this year is immense, and greater than ever before. In California, during the early emigration, men would squander a vast deal of money in quinine, which was not to be had for a song, prescribing for themselves, and turning medicine taking into a kind of self-indulgence. Undoubtedly, quinine, if it does no more, alleviates fever and ague; but a man who goes on taking it all his life and neglecting natural and ordinary precautions, will ruin his physical constitution and misspend his money.

There was a time during the penisular campaign, when many of the troops on the Chickahominy, under the advice of the surgeons, regularly took their whisky and quinine every morning, to ward off the supposed tendency in that locality to "fevers."—*Medical and Surgical Reporter.*

